# A Feeling for Numbers: Shared Metric for Symbolic and Tactile Numerosities

**DOI:** 10.3389/fpsyg.2013.00007

**Published:** 2013-01-25

**Authors:** Florian Krause, Harold Bekkering, Oliver Lindemann

**Affiliations:** ^1^Donders Institute for Brain, Cognition and Behaviour, Radboud University NijmegenNijmegen, Netherlands; ^2^Division of Cognitive Science, University of PotsdamPotsdam, Germany

**Keywords:** number cognition, tactile perception, finger counting

## Abstract

Evidence for an approximate analog system of numbers has been provided by the finding that the comparison of two numerals takes longer and is more error-prone if the semantic distance between the numbers becomes smaller (so-called numerical distance effect). Recent embodied theories suggest that analog number representations are based on previous sensory experiences and constitute therefore a common magnitude metric shared by multiple domains. Here we demonstrate the existence of a cross-modal semantic distance effect between symbolic and tactile numerosities. Participants received tactile stimulations of different amounts of fingers while reading Arabic digits and indicated verbally whether the amount of stimulated fingers was different from the simultaneously presented digit or not. The larger the semantic distance was between the two numerosities, the faster and more accurate participants made their judgments. This cross-modal numerosity distance effect suggests a direct connection between tactile sensations and the concept of numerical magnitude. A second experiment replicated the interaction between symbolic and tactile numerosities and showed that this effect is not modulated by the participants’ finger counting habits. Taken together, our data provide novel evidence for a shared metric for symbolic and tactile numerosities as an instance of an embodied representation of numbers.

## Introduction

It has been argued that numbers are cognitively represented in an approximate and analog manner (e.g., Dehaene et al., 1993). Main evidence for this notion comes from the so-called *numerical distance effect* (Moyer and Landauer, 1967). When participants are asked to perform a magnitude judgment (i.e., compare two numbers by their semantic size) responses are slower, when the semantic distance between the two numbers is small (e.g., 2 vs. 3), compared to when the semantic distance is large (e.g., 1 vs. 4; Gallistel and Gelman, 1992). This effect of the numerical distance has been consistently explained by a representational overlap of neighboring numbers on a hypothetical analog mental continuum of numerical magnitudes (e.g., Restle, 1970; Dehaene and Changeux, 1993). That is, a particular number does not only activate the representation of exactly this number, but also the representation of the numbers next to it. Consequently, the further apart two numbers are, the less do they activate each other and the easier it is to discriminate between them. Support for this idea is also provided by studies on human and non-human cortical activations in response to numerosity information that demonstrated the existence of number-sensitive neurons with overlapping tuning curves in macaque (Nieder and Miller, 2003) as well as in the human parietal cortex (Piazza et al., 2004). Although the existence of an analog representation in humans and animals is very established, the origin and nature of this specific semantic representation of magnitude information is controversially debated (see e.g., Cohen Kadosh and Walsh, 2009).

In modern psycholinguistic research, several authors emphasized the idea of embodied cognition (Wilson, 2002), which basically holds that each semantic representation is grounded in previous sensorimotor experiences and therefore closely linked to low-level perceptual and motor codes (Glenberg and Kaschak, 2002; Barsalou, 2008; Fischer and Zwaan, 2008). Interestingly, the role of embodied representations has also been recently discussed in the context of number processing. For instance, recent research has shown that the perception of abstract numerical stimuli has a direct influence on response selection (Daar and Pratt, 2008) as well as movement generation (Vicario, 2012), demonstrating a close link between numerical concepts and action. It has been speculated that numerical magnitude information becomes meaningful only when it can be somehow mapped to concrete bodily experiences with size and magnitude in everyday life (Andres et al., 2008; Lindemann et al., 2009). A similar important role of size-related sensorimotor representations for numbers has been suggested by a recent theory on magnitude representations proposed by Walsh (2003), which assumes the existence of a shared generalized representation of magnitude. That is, numbers are thought to be processed by a single system which simultaneously codes for size-related information from other cognitive domains like, for instance, sensory and motor representations of physical size or temporal duration. Evidence for this notion comes from behavioral studies showing interferences between numbers and other types of magnitude information, such as the physical size of number symbols (Tzelgov et al., 1992), the perceived time (Oliveri et al., 2008), the perceived size of an object (Badets et al., 2007), and the aperture size while object grasping (Lindemann et al., 2007).

Another observation often interpreted as evidence for an embodied representation of numbers is the existence of a strong association of fingers and numbers in most adults. This association is probably resulting from the habit to use fingers while counting (Lindemann et al., 2011; Bender and Beller, 2012; Moeller et al., 2012). For instance, in Italian adults this association has been demonstrated by a facilitation to respond to numbers 1 to 5 with the fingers of the right hand, and to numbers 6 to 10 with the fingers of the left hand (a mapping congruent to the prototypical finger counting strategy of the participants; Di Luca et al., 2006), as well as by a facilitation to judge if a number is smaller or larger than five when primed with a finger configuration compatible to the individual’s counting strategy (Di Luca and Pesenti, 2008). In addition to this, we know that the preference to start counting with the left or with the right hand varies strongly between individuals and is independent of handedness (Lindemann et al., 2011). Interestingly, manual counting habits, like the individual finger-number associations and starting preferences have been shown to affect symbolic number processing in adults, even if the use of fingers is not required (Fischer, 2008; Domahs et al., 2010). For instance, Fischer (2008) showed that the association of numbers with a spatial response (SNARC effect; Dehaene et al., 1993) was strongly affected by whether participants started to count on their right or left hand. Only for participants who started counting on their left hand a SNARC effect could be observed. In another study Domahs et al. (2010) investigated finger-based sub-base-five effects in an Arabic number comparison task in three different groups – German deaf signers, German hearing adults, and Chinese hearing adults. Their results revealed that sub-base-five effects were larger in the two German groups which use a sub-base-five finger counting system, compared to the Chinese group which uses a sub-base-10 finger counting system. Taken together, these studies speak for an important role of finger representations for the processing of symbolic numerical information.

While an increasing amount of studies investigated the cognitive effects of the finger-number associations, until today only few studies have examined tactile or haptic numerosity processing as such. As we know from recent experiments on tactile and haptic perception (Riggs et al., 2006; Plaisier et al., 2009; Plaisier and Smeets, 2011), tactile numerosity perception seems to be based on the same distinct cognitive processes as the enumeration of visual items (Atkinson et al., 1976). For instance, Riggs et al. (2006) stimulated the fingertips of their participants and asked them to name the number of stimulated fingers. The authors found that judgments were based on serial counting processes if more than three fingers were stimulated, since enumeration became more error-prone and slower with increasing set-size. In contrast, however, for small numerosities (i.e., less than four fingers) tactile enumeration was fast, effortless, and highly accurate (Riggs et al., 2006; Plaisier and Smeets, 2011; but see also Gallace et al., 2008) – a phenomenon well known from vision research and called “subitizing” (Kaufman et al., 1949). Recently, support for subitizing has also been demonstrated for active touch and the haptic exploration of the amount of objects in the hand (Plaisier et al., 2009; Plaisier and Smeets, 2011). That is, there is increasing evidence that numerosity perception in the tactical and in the visual modality share the same processes. These findings suggest that all sensory numerosity information are represented by the same modality-independent magnitude system.

Taking into account the embodied view on cognition (e.g., Wilson, 2002; Barsalou, 2008) and the idea of a single generalized metric for magnitudes (Walsh, 2003), one might speculate that tactile numerosity processing is based on the very same analog magnitude representation that is activated when reading symbolic numbers or solving arithmetic problems. Surprisingly, however, very little is known about the relationship and the commonalities between tactile and symbolic numbers. We assumed that tactile numerosity judgments are based on the same analog representations as involved in symbolic number processing irrespective of differences in format and modality. To examine this hypothesis, we made use of the numerical distance effect (Moyer and Landauer, 1967). We conducted two experiments, in which participants received tactile stimulations on their fingers of the left or right hand while reading an Arabic digit. The participants’ task was to indicate as fast as possible whether the visually presented number matched the amount of stimulated fingers. If both tactile and symbolic numerosities are indeed mapped onto the same analog magnitude metric, we expected to observe a cross-modal numerosity distance effect reflected by an inverse linear relation between the judgment latencies in the magnitude comparison task and the semantic distance between the to-be compared numerosities. Crucially, we used a same-different task, and not a magnitude comparison task. That is, if alternatively, symbolic and tactile numerosities activate different analog magnitude representations or same-different comparisons find place on verbal codes, a modulation of the response latencies as a function of the semantic distance is not expected (cf. Van Opstal and Verguts, 2011; Defever et al., 2012).

Moreover, if the acquired associations between fingers and number modulate adults’ processing of symbolic numerosity information, one might expect that counting habits also affect the enumeration or perception of numbers in the subitizing range. We therefore aimed to explore additionally the influence of finger counting habits on the tactile perception of numerosities and their comparison with symbolic numbers. To do so, we used an adapted version of the finger counting questionnaire of Lindemann et al. (2011) to classify the starting preference of our participants and tested whether detection times or cross-modal numerosity distance effects are modulated by these habits.

## Experiment 1

The aim of the first experiment was to investigate if tactile numerosities are mapped to the same analog representation of numerical magnitude as symbolic numerosities, as expected by the notion of a generalized magnitude system (Walsh, 2003). Participants had to verbally indicate if tactile presented numerosities were identical or different to visually presented Arabic digits. We expected to find a cross-modal semantic distance effect in the numerosity judgments reflected by longer response times when comparing tactile and symbolic numerosities that are close in distance. Furthermore, if finger counting habits affect this analog representation of numerical magnitude, both starting hand preferences as well as specific finger preferences should modulate a cross-modal numerosity distance effect.

### Method

#### Participants

Twenty-four students (five male, two left-handed) between 17 and 33 years of age (mean = 21.33, SD = 3.61) participated in the study in return of €5 or credit points. All of them reported to have normal or corrected-to-normal vision.

#### Setup

Participants were seated in front of a table with a computer screen (viewing distance approximately 60 cm) and two custom-made tactile stimulation devices (one for each hand; see also van Ede et al., 2010), each consisting of five piezoelectric Braille cells (Metec AG, Stuttgart, Germany). Each Braille cell had eight pins, arranged in two groups of four, which can be raised and lowered for about 1 mm. The tactile stimulation devices were each placed into a wooden, sound-shielded box on the table in front of the participant, such that he or she could neither see the hands being stimulated, nor hear mechanical noise from the stimulation. The orientation of the tactile stimulation devices within the boxes was such that participants could place their hands in a comfortable horizontally oriented resting position. A dynamic microphone and a custom-made voice-key device was used to record voice-onsets. The experiment was controlled using custom-made software. The experimenter was seated out of the participants’ vision at a second table and used a keyboard to enter which verbal response was given.

#### Material

Visual target stimuli comprised the digits “1,” “2,” “3,” and “4” presented in a light gray color in front of a dark background. Tactile target stimuli consisted of the simultaneous stimulation of one to four fingers of either the left or right hand. To examine the impact of the counting habits, always one to four suggestive fingers were stimulated starting with either the thumb or pinkie. That is, there were in total eight patterns of stimulation for each hand: four medial finger sets in which the number of stimulated fingers was started with the thumb (1 = [Thumb], 2 = [Thumb, Index Finger], 3 = [Thumb, Index Finger, Middle Finger], 4 = [Thumb, Index Finger, Middle Finger, Ring Finger]) and four lateral finger sets starting with the pinkie. (1 = [Pinkie], 2 = [Pinkie, Ring Finger], 3 = [Pinkie, Ring Finger, Middle Finger], 4 = [Pinkie, Ring Finger, Middle Finger, Index Finger]). Depending on the reported finger counting preferences each stimulation pattern could be classified as either finger counting compatible or incompatible.

Individual finger counting habits and starting preference of each participant were determined by a finger counting questionnaire (Lindemann et al., 2011).

#### Procedure

Each trial began with the presentation of a fixation cross for 500 ms, followed by the simultaneous onset of the visual and tactile target stimuli. Tactile stimulation consisted of a repeated switching between raised (20 ms) and lowered (30 ms) states of all pins. Participants were instructed to decide whether the amount of stimulated fingers was equal or different to the numerical size of the visually presented digit. Responses were given verbally by uttering “Tee” (when the numerosities were identical) or “Toh” (when the numerosities were different). Since voice onset times served as decision time measures, we decided to use verbal utterances for which the first transient is phonologically the same. The target stimuli (tactile and visual) disappeared as soon as a verbal response was given and a blank screen was presented for a variable time between 1000 and 1500 ms. No feedback was given for erroneous responses. The next trial started after the experimenter classified given responses.

#### Design

The experiment consisted of four blocks. Each block contained 128 trials (two repetitions of all possible combinations of eight stimulation patterns on two hands and four visually presented digits). All trials were presented in randomized order. The duration of the experiment was approximately 30 min.

### Results

#### Finger counting preferences

The analysis of the finger counting questionnaire yielded that 58.3% of the participants preferred to start counting with their left and 41.7% with their right hand. Twenty-one participants reported a typical unimanual counting pattern for Western subjects and to start counting with the thumb. One participant reported a counting pattern that could not be classified according to existing categories of starting hand and preferred finger sequence (cf. Lindemann et al., 2011) and therefore had to be excluded from the analysis. The other two participants started counting with the pinkie and counted in successive order to the thumb. The reported finger counting pattern was used to classify the stimulated set of fingers into counting habit compatible and incompatible sets for all participants. That is, for 21 participants the medial fingers (thumb, index finger, middle finger, and ring finger) were classified as counting habit compatible and the lateral fingers (pinkie, ring finger, middle finger, and index finger) as counting habit incompatible, while for two participants the lateral fingers were classified as counting habit compatible and the medial fingers as counting habit incompatible.

#### Numerosity comparisons

Responses that deviated more than three SD from the mean response time of each participant (anticipatory responses: 0.04%; slow responses: 1.63%) were excluded from further analysis. Erroneous responses occurred in 7.37% of all remaining trials and were excluded from the response time analysis.

Median response times and errors were each entered in a separate repeated measures ANOVA with the within-subject factors Semantic Distance (0, 1, 2, 3), Set of Fingers (counting habit compatible, counting habit incompatible), Hand (left, right), and the between-subject factor Starting Hand (left, right). Reported degrees of freedom for the *F* statistics were Huynh–Feldt corrected, when necessary.

**Figure 1:**
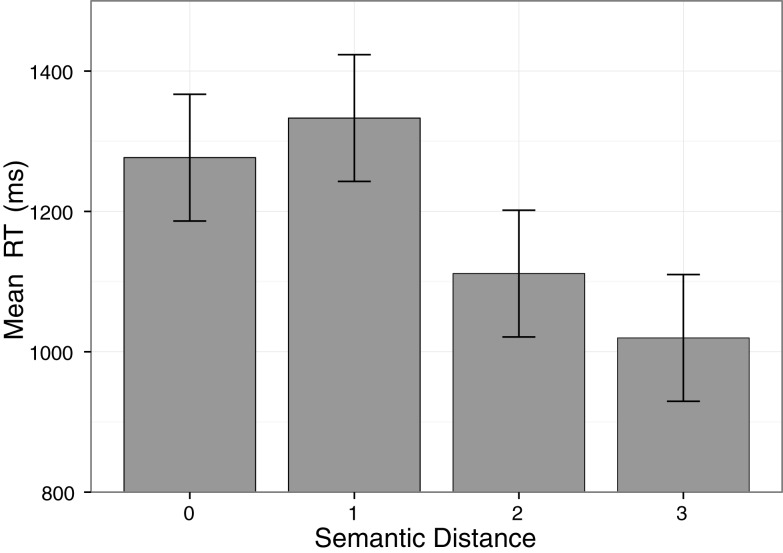
**The cross-modal semantic distance effect between tactile and symbolic numerosities**. The larger the semantic distance between both numerosities, the shorter the mean response time. Error bars represent 95% within-subject confidence intervals (cf. Loftus and Masson, 1994).

In line with our hypothesis, the reaction time analysis revealed a significant main effect of Semantic Distance, *F*(1.84, 38.73) = 25.03, *p* < 0.001, $\eta_{p}^{2}$ = 0.54, showing an interaction of tactile and symbolic numerosities. Responses were faster for a numerical distance between tactile and symbolic numerosity of 3 compared to a distance of 2, *t*(22) = −4.21, *p* < 0.001, as well as for a distance of 2 compared to a distance of 1, *t*(22) = −6.06, *p* < 0.001. There was no significant difference between a distance of 1 and a distance of 0 (same numerosity in both modalities), *t*(22) = 1.52, *p* = 0.14 (see Figure 1). There was a significant main effect of Set of Fingers, *F*(1, 21) = 11.55, *p* < 0.01, $\eta_{p}^{2}$ = 0.36, reflecting shorter reaction times when stimulating the counting habit compatible fingers (i.e., for most participants starting from thumb to pinkie; 1131 ms), compared to the counting habit incompatible fingers (i.e., for most participants starting from pinkie to thumb; 1185 ms). The main effect of Hand did not reach significance, *F*(1, 21) = 0.41, *p* = 0.53, $\eta_{p}^{2}$ = 0.02. Interestingly, the factors Semantic Distance and Set of Fingers interacted significantly, *F*(3, 63) = 8.80, *p* < 0.001, $\eta_{p}^{2}$ = 0.30. *Post hoc* *F*-tests showed a stronger effect of Semantic Distance for the counting habit incompatible finger stimulations, *F*(2.20, 46.09) = 26.25, *p* < 0.001, $\eta_{p}^{2}$ = 0.56, than for the compatible stimulations, *F*(2.01, 42.17) = 15.09, *p* < 0.001, $\eta_{p}^{2}$ = 0.42. No significant effects were observed for the interactions Semantic Distance × Hand, *F*(2.41, 50.61) = 2.41, *p* = 0.09, $\eta_{p}^{2}$ = 0.10, Semantic Distance × Starting Hand, *F*(3, 63) = 0.02, *p* = 1.0, $\eta_{p}^{2}$ = 0.001, the Set of Fingers × Hand, *F*(1, 21) = 0.04, *p* = 0.84, $\eta_{p}^{2}$ = 0.002, Set of Fingers × Starting Hand, *F*(3, 63) = 0.20, *p* = 0.66, $\eta_{p}^{2}$ = 0.01, Hand × Starting Hand, *F*(1, 21) = 1.08, *p* = 0.31, $\eta_{p}^{2}$ = 0.05, Semantic Distance × Set of Fingers × Hand, *F*(3, 63) = 0.61, *p* = 0.61, $\eta_{p}^{2}$ = 0.03, Semantic Distance × Set of Fingers × Starting Hand, *F*(3, 63) = 0.32, *p* = 0.81, $\eta_{p}^{2}$ = 0.02, Semantic Distance × Hand × Starting Hand, *F*(3, 63) = 1.90, *p* = 0.14, $\eta_{p}^{2}$ = 0.08, Set of Fingers × Hand × Starting Hand, *F*(1, 21) = 0.06, *p* = 0.81, $\eta_{p}^{2}$ = 0.003, and Semantic Distance × Set of Fingers × Hand × Starting Hand, *F*(3, 63) = 0.06, *p* = 0.98, $\eta_{p}^{2}$ = 0.003.

The error analysis also revealed a significant main effect of Semantic Distance, *F*(2, 42) = 21.02, *p* < 0.001, $\eta_{p}^{2}$ = 0.50. That is, participants made fewer errors for a numerical distance between tactile and symbolic numerosity of 3 compared to a distance of 1, *t*(22) = −4.58, *p* < 0.001, as well as for a distance of 2 compared to a distance of 1, *t*(22) = −5.10, *p* < 0.001. There was no significant difference between a distance of 1 and a distance of 0, *t*(22) = 0.76, *p* = 0.46. No main effects were observed for the factors Set of Fingers, *F*(1, 21) = 0.02, *p* = 0.89, $\eta_{p}^{2}$ = 0.001, and Hand, *F*(1, 21) = 1.86, *p* = 0.19, $\eta_{p}^{2}$ = 0.08. There were no significant effects for the interactions Semantic Distance × Set of Fingers, *F*(1.52, 32.14) = 0.39, *p* = 0.63, $\eta_{p}^{2}$ = 0.02, Semantic Distance × Hand, *F*(2.27, 47.66) = 1.94, *p* = 0.15, $\eta_{p}^{2}$ = 0.09, Semantic Distance × Starting Hand, *F*(3, 63) = 0.44, *p* = 0.73, $\eta_{p}^{2}$ = 0.02, the Set of Fingers × Hand, *F*(1, 21) = 0.16, *p* = 0.69, $\eta_{p}^{2}$ = 0.008, Set of Fingers × Starting Hand, *F*(1, 21) = 0.22, *p* = 0.65, $\eta_{p}^{2}$ = 0.01, Hand × Starting Hand, *F*(1, 21) = 0.11, *p* = 0.74, $\eta_{p}^{2}$ = 0.005, Semantic Distance × Set of Fingers × Hand, *F*(1.87, 39.32) = 0.28, *p* = 0.75, $\eta_{p}^{2}$ = 0.01, Semantic Distance × Set of Fingers × Starting Hand, *F*(3, 63) = 0.42, *p* = 0.74, $\eta_{p}^{2}$ = 0.02, Semantic Distance × Hand × Starting Hand, *F*(3, 63) = 1.71, *p* = 0.17, $\eta_{p}^{2}$ = 0.08, Set of Fingers × Hand × Starting Hand, *F*(1, 21) = 0.3.70, *p* = 0.07, $\eta_{p}^{2}$ = 0.15, and Semantic Distance × Set of Fingers × Hand × Starting Hand, *F*(3, 63) = 1.82, *p* = 0.15, $\eta_{p}^{2}$ = 0.08.

### Discussion

As hypothesized, we found a cross-modal numerosity distance effect in the magnitude comparison task when participants were instructed to compare tactile presented numerosities with symbolically presented numerosities. That is, participants became faster and made fewer errors to judge the difference between tactile and symbolic numerosities, when the semantic distance between both numerosities was increased. This finding suggests that tactile numerosities are mapped to the same analog representation of magnitude as symbolic numerosities.

While starting preferences did not modulate the cross-modal distance effect, it was modulated by the set of fingers stimulated. Interestingly, the effect was stronger for counting habit incompatible finger sets than for counting habit compatible finger sets. This appears counter-intuitive as one would have expected the exact opposite pattern if finger representations were connected to an analog numerical magnitude representation, that is, a stronger effect for counting habit compatible finger sets. Furthermore, it has to be noticed that the vast majority of our subjects showed a prototypical Western finger counting habit (Lindemann et al., 2011) and started counting with the medial fingers from thumb to pinkie. Consequently, the dissociation between counting habit compatible and incompatible finger sets goes in the present study along with the dissociation between medial (i.e., starting from thumb) and lateral fingers (i.e., starting from pinkie), which seems to be a problematic confound for the interpretation of our findings. Consequently, it remains unclear if the differences between the stimulated finger sets and the modulation of the numerosity distance effect were driven by differences in the finger counting preferences or whether they merely reflected differences in the hand physiology between the medial and lateral finger sets and resulting differences in touch acuity and cortical representation (cf. Elbert et al., 1995). To be more precise, a more developed cortical representation of the medial fingers could account for a faster and more precise detection of a tactile stimulation of these fingers, compared to the lateral fingers with a less developed cortical representation. To specifically investigate the influence of finger counting habits in our setting, independent of such physiological differences, we conducted a second experiment in which the same set of fingers was sequentially stimulated. Importantly, the type of sequence and direction of the tactile stimulations, not the set of fingers, defined the compatibility with finger counting habits.

## Experiment 2

The second experiment tests a potential influence of finger counting habits for the detection and representation of tactile numerosities. Since it cannot be excluded that the effect of the set of fingers in Experiment 1 might be driven by physiological differences, Experiment 2 aimed to introduce finger counting compatible and incompatible tactile numerosities while keeping the set of stimulated fingers constant. This has been achieved by sequential stimulations in two different directions; either starting from the thumb or starting from the ring finger. If finger counting habits influence the analog representation of numerical magnitude, participants that start counting with the thumb are expected to show a different cross-modal numerosity distance effect if the sequence of stimulation was not compatible to their direction of counting.

### Method

#### Participants

Twenty-eight students (eight male, one left-handed) between 18 and 25 years of age (mean = 20.07, SD = 2.37) participated in the study in return of €5 or credit points. None of them participated in Experiment 1. All of them reported to have normal or corrected-to-normal vision.

#### Setup and material

The setup and material were identical to that of Experiment 1. The experiment was controlled using the software *Expyriment* (Krause and Lindemann, 2012). Participants were asked to indicate starting preference and specific finger counting habits (cf. Lindemann et al., 2011).

#### Procedure and design

The procedure and design were similar to Experiment 1, with two exceptions. First, tactile stimuli consisted of a stimulation of one to four fingers (1 = [Thumb] to 4 = [Thumb, Index Finger, Middle Finger, Ring Finger]). Crucially, all fingers were sequentially stimulated in two different directions: a forward direction, starting from the thumb, and a backward direction, starting from the last finger ending with the thumb. Second, the onset of a visual stimulus was equivalent to the offset of the tactile stimulation. This was done to ensure that response times were not confounded with differences in sequence length (e.g., when seeing the digit 1, a response could already be given after one finger is stimulated, while when seeing a 4, one would need to wait until all four finger have been stimulated). Tactile stimulation always started with the stimulation of a single finger. After each 100 ms the next finger in the sequence was added to the stimulation. When all fingers were added the stimulation continued on all fingers until a total stimulation time of 600 ms was reached.

### Results

#### Finger counting preferences

The analysis of finger counting habits yielded that 57.1% of the participants preferred to start counting with their left and 42.9% with their right hand. Crucially, all participants reported to start counting with the thumb.

#### Numerosity comparisons

Erroneous responses (8.46%) as well as responses that deviated more than three SD from individual mean response times (only fast responses: 0.22%) were excluded from the response time analysis of the numerosity comparisons. Since all investigated participants started counting with the thumb, tactile stimulations in forward direction could be considered as finger counting compatible and backward stimulations as finger counting incompatible. Errors and median response times were each entered into a separate repeated measures ANOVA with the within-subject factors Semantic Distance (0, 1, 2, 3), Direction of Stimulation (finger counting compatible, finger counting incompatible), Hand (left, right), and the between-subject factor Starting Hand (left, right). Reported degrees of freedom for the *F* statistics were Huynh–Feldt corrected when necessary.

**Figure 2:**
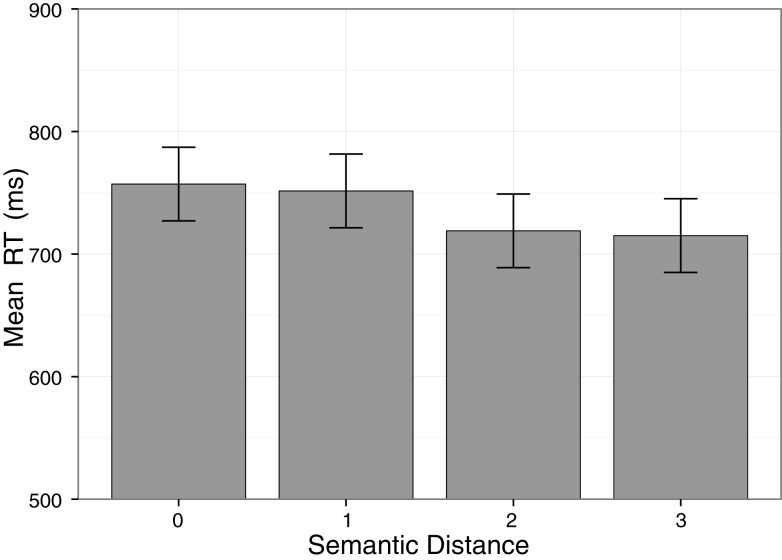
**The cross-modal semantic distance effect between tactile and symbolic numerosities for sequential tactile stimulation**. Mean response times are significantly shorter for a large semantic distance of 2 and 3 than for a small semantic distance of 1. Error bars represent 95% within-subject confidence intervals (cf. Loftus and Masson, 1994).

As in Experiment 1, the response time analysis revealed a significant main effect of Semantic Distance, *F*(2.01, 52.21) = 8.09, *p* < 0.001, $\eta_{p}^{2}$ = 0.24, confirming our main hypothesis of an interaction of tactile and symbolic numerosities. That is, responses were faster for a numerical distance between a tactile and symbolic stimulus of 3 compared to a distance of 1, *t*(27) = −4.73, *p* < 0.001, as well as for a distance of 2 compared to a distance of 1, *t*(27) = −4.36, *p* < 0.001. There was no significant difference between a distance of 1 and a distance of 0 (same numerosity in both modalities), *t*(27) = −0.29, *p* = 0.77 (see also Figure 2). There was only a trend for an effect of Direction of Stimulation, *F*(1, 26) = 4.10, *p* = 0.053, $\eta_{p}^{2}$ = 0.14, with descriptively slightly shorter reaction times for finger counting compatible stimulation sequence (732 ms) compared to incompatible stimulations (739 ms). That is, in contrast to Experiment 1, we did not observe a reliable advantage of finger counting compatible stimulations. No main effect of the factor Hand was observed, *F*(1, 26) = 0.15, *p* = 0.70, $\eta_{p}^{2}$ = 0.01. Importantly, there was no interaction between the factors Semantic Distance and Direction of Stimulation, *F*(3, 78) = 0.12, *p* = 0.95, $\eta_{p}^{2}$ = 0.01, showing that, unlike in Experiment 1, the numerical distance effect was not modulated by finger counting compatibility. No significant effects were observed for the interactions Semantic Distance × Hand, *F*(3, 78) = 1.23, *p* = 0.31, $\eta_{p}^{2}$ = 0.05, Semantic Distance × Starting Hand, *F*(3, 78) = 0.75, *p* = 0.52, $\eta_{p}^{2}$ = 0.03, Direction of Stimulation × Hand, *F*(1, 26) = 0.12, *p* = 0.73, $\eta_{p}^{2}$ = 0.01, Direction of Stimulation × Starting Hand, *F*(1, 26) = 0.16, *p* = 0.69, $\eta_{p}^{2}$ = 0.01, Hand × Starting Hand, *F*(1, 26) = 0.12, *p* = 0.73, $\eta_{p}^{2}$ = 0.01, Semantic Distance × Direction of Stimulation × Hand, *F*(3, 78) = 0.83, *p* = 0.48, $\eta_{p}^{2}$ = 0.03, Semantic Distance × Direction of Stimulation × Starting Hand, *F*(3, 78) = 1.07, *p* = 0.37, $\eta_{p}^{2}$ = 0.04, Semantic Distance × Hand × Starting Hand, *F*(3, 78) = 0.85, *p* = 0.47, $\eta_{p}^{2}$ = 0.03, Direction of Stimulation × Hand × Starting Hand, *F*(1, 26) = 3.35, *p* = 0.08, $\eta_{p}^{2}$ = 0.11, and Semantic Distance × Direction of Stimulation × Hand × Starting Hand, *F*(3, 78) = 2.30, *p* = 0.08, $\eta_{p}^{2}$ = 0.08.

The error analysis revealed a significant main effect of Semantic Distance, *F*(2.28, 59.17) = 30.45, *p* < 0.001, $\eta_{p}^{2}$ = 0.54, with fewer errors for a distance of 3 compared to a distance of 1, *t*(27) = −4.07, *p* < 0.001, as well as a distance of 2 compared to a distance of 1, *t*(27) = −4.27, *p* < 0.001. The difference between a distance of 1 and a distance of 0 was significant as well, *t*(27) = −2.93, *p* < 0.01. No significant main effects were observed for the factors Direction of Stimulation, *F*(1, 26) = 1.58, *p* = 0.22, $\eta_{p}^{2}$ = 0.06, and Hand, *F*(1, 26) = 0.44, *p* = 0.51, $\eta_{p}^{2}$ = 0.02. The 4-way interaction Semantic Distance × Direction of Stimulation × Hand × Starting Hand was significant, *F*(3, 78) = 3.28, *p* < 0.05, $\eta_{p}^{2}$ = 0.11. Since our hypotheses are independent from this observed 4-way interaction between all factors, we did not further analyze and interpret this complex effect. There were no significant effects for the interactions Semantic Distance × Direction of Stimulation, *F*(2.27, 59.07) = 1.39, *p* = 0.28, $\eta_{p}^{2}$ = 0.05, Semantic Distance × Hand, *F*(2.26, 68.19) = 1.07, *p* = 0.36, $\eta_{p}^{2}$ = 0.04, Semantic Distance × Starting Hand, *F*(3, 78) = 0.13, *p* = 0.94, $\eta_{p}^{2}$ = 0.01, the Direction of Stimulation × Hand, *F*(1, 26) = 1.81, *p* = 0.19, $\eta_{p}^{2}$ = 0.07, Direction of Stimulation × Starting Hand, *F*(1, 26) = 0.74, *p* = 0.40, $\eta_{p}^{2}$ = 0.03, Hand × Starting Hand, *F*(1, 26) = 0.34, *p* = 0.56, $\eta_{p}^{2}$ = 0.01, Semantic Distance × Direction of Stimulation × Hand, *F*(2.43, 63.12) = 2.09, *p* = 0.12, $\eta_{p}^{2}$ = 0.07, Semantic Distance × Direction of Stimulation × Starting Hand, *F*(3, 78) = 0.62, *p* = 0.61, $\eta_{p}^{2}$ = 0.02, Semantic Distance × Hand × Starting Hand, *F*(3, 78) = 0.07, *p* = 0.98, $\eta_{p}^{2}$ = 0.003, and Direction of Stimulation × Hand × Starting Hand, *F*(1, 26) = 0.39, *p* = 0.54, $\eta_{p}^{2}$ = 0.02.

### Discussion

Experiment 2 confirmed the finding of the cross-modal numerosity distance effect from Experiment 1. Again, the effect was present in both response times and error rates.

However, the cross-modal numerosity distance effect was not modulated by any finger counting preferences (Starting Hand or Direction of Stimulation), as would have been expected, if counting habits influence the analog representation of numerical magnitude. We interpret this as evidence that a common metric shared by the representation of tactile and symbolic numerosity information reflects a magnitude representation that is independent of finger representations and analog numerosity representations acquired while learning to count with the fingers.

In contrast to Experiment 1, in which finger counting compatibility led to faster responses, but was confounded with hand physiology, neither the stimulation direction nor the starting preference significantly influenced the perception of the tactile stimulus. While there was a trend for a main effect of Direction of Stimulation no main effect for Starting Hand could be observed. Thus, while counting habits do not influence a shared magnitude representation, they might have a marginal influence on the perception of a tactile stimulus.

## General Discussion

The current study demonstrates an interference between fingers and numbers on the level of analog numerical magnitude representations. In two experiments we investigated the relation between tactile and symbolic numerosities, and the influence of finger counting habits thereon. Our data provide first evidence for the existence of a cross-modal semantic distance effect in participants comparing tactile presented numerosities with symbolically presented numerosities. More specifically, responses were faster and less error-prone when judging two distant numerosities (e.g., 1 and 4) than when judging two close-by numerosities (e.g., 1 and 2).

Importantly, all numerosities used in the current study were within the range of subitizing and are thus assumed to be perceived directly and accurately without relying on a serial counting process (Riggs et al., 2006; Plaisier and Smeets, 2011). We can therefore assume that our results (at least in Experiment 1, where the stimulation was non-sequential) are not mediated by verbally counting the stimulated fingers. Rather, since the numerical distance effect has been consistently interpreted to reflect a representational overlap between neighboring items on an analog continuum (Moyer and Landauer, 1967; Restle, 1970), our results suggest that tactile presented numerosities were automatically mapped onto the same analog representation as symbolic numerosities. This interpretation receives further support by the fact that participants made a same-different judgment (and not a magnitude judgment), as it has been shown that the numerical distance effect resulting from a same-different judgment crucially depends on overlapping analog representations (Van Opstal and Verguts, 2011). Thus, the fact that we find a numerical distance effect allows us to exclude the possibility that the comparison of the tactile and symbolic numerosities was done by merely comparing verbal codes (since this would not have led to a distance effect). It is also unlikely that the magnitude representation of both numerosities was not activated directly, but through a preceding verbal code, since it has been shown that already preschoolers use surface features of numerical stimuli instead of a magnitude representation to solve a same-different judgment, when available (Defever et al., 2012). This means that if a verbal code preceded a magnitude representation in our setting, judgments could have already been solved on this more direct verbal level, without the need for a more abstract representation of the numerical magnitude. Crucially, again, a same-different judgment on the basis of such verbal codes would not have led to a numerical distance effect. Taken together, the current finding suggests that tactile and numerical numerosities share a common analog representation of numerical magnitude.

The finding of a cross-modal numerosity distance effect is in line with the notion of a generalized magnitude system (Walsh, 2003), which hypothesizes that the brain processes general magnitude information according to a shared metric, independent from the domain this magnitude information comes from. In our study, magnitude information from two different modalities (tactile, visual) and with two different notations (symbolic, non-symbolic) had to be processed and compared. The observation that the judgment latencies and accuracies depended on the cross-modal numerosity distance suggests that both types of numerosity information were mapped onto the same analog magnitude representation which were then utilized for the actual cognitive comparison. It has to be furthermore mentioned that the current study is focusing on the processing of small numerosities. It is therefore unclear whether visual and tactile numerosities share also cognitive codes for larger numbers. Taking into account the possibility that common representations are shaped while using the fingers to count, it is an important open question for further research whether these cross-domain associations are also present for numerosities larger 10.

The conclusion that processing of sensory and symbolic numerosity information leads to an activation of common analog codes supports the idea of embodied numerosities. The embodied cognition view claims that abstract cognitive concepts are “grounded” in sensorimotor experiences (Barsalou, 2008). That is, the content of abstract concepts, like numbers, is assumed to become meaningful by being coupled to bodily representations (Lindemann et al., 2009). Here, the cross-modal semantic distance effect reveals a direct relationship between tactile and abstract numerosities and the presence of a magnitude metric shared by both modalities. Representations of sensory experiences about size and numerosity might this way provide a grounding for the meaning of symbolic numbers and might therefore play a crucial role in the development of number concepts.

While we cannot entirely exclude that finger counting habits are responsible for the differences in the numerical distance effect between the sets of fingers found in Experiment 1, our data does also not provide any evidence for this. We observed a stronger numerical distance effect for the fingers which are not used to represent the numerosities during counting. However, if finger representations were indeed connected to an analog numerical magnitude representation, one would have expected the opposite, namely, a stronger numerical distance effect for those fingers compatible to this representation. Considering furthermore that no influence of finger counting habits on the numerical judgments could be found when the same set of fingers was stimulated in different sequential orders (Experiment 2), it seems very likely that physiological differences between the medial and lateral sets of fingers were responsible for the observed differences in the judgment latencies of Experiment 1.

In contrast to our study, some previous studies reported an influence of finger counting habits on the processing of symbolic numbers (e.g., Di Luca et al., 2006; Di Luca and Pesenti, 2008). The question arises therefore why finger counting habits did not affect the cross-modal numerosity comparison as investigated in the present paradigm. First, it is important to note that most of the existing literature demonstrated associations between finger patterns and numbers by means of a faster detection or stronger number activations for canonical finger patterns. These effects might be mediated by a perceptual familiarity of canonical finger patterns. While we observed a similar pattern of facilitation in Experiment 2 where stimulation sequences compatible with the participants’ finger counting pattern were detected slightly faster and processed more fluently, this effect was, however, not statistically significant. Second, the current study is one of the first to investigate the influence of finger counting habits on an analog representation of numerical magnitude in the subitizing range. Following the literature on subitizing, this should have resulted in a very automatic and fast activation of the number concept (Kaufman et al., 1949). The absence of any influence of finger counting habits under these circumstances suggests that differently preferred patterns of fingers are not differently coupled to an analog representation of numerical magnitude. Typical finger counting patterns might instead constitute an additional independent numerical representation (see also Moeller et al., 2012 for a similar proposal) and represent verbally and perceptually mediated associations between postures and number meaning that are acquired while learning to count.

While the presence of cross-modal numerical distance effects supports the view of an embodied representation of numerical magnitude, we argue that the fact that this phenomenon is independent of acquired finger counting preferences shows that finger counting postures serve as the function of motor symbols and reflect probably the individuals’ cognitive strategy to offload numerical information (Lindemann and Krause, 2012).

Taken together, the current study provides evidence for a shared metric for tactile and symbolic numerosities, as an instance of an embodied representation of numbers. Crucially, the underlying analog representation of numerical magnitude information appeared to be independent from finger representations.

## Conflict of Interest Statement

The authors declare that the research was conducted in the absence of any commercial or financial relationships that could be construed as a potential conflict of interest.

## References

[B1] Andres M. Olivier E. Badets A. (2008). Actions, words, and numbers: a motor contribution to semantic processing? Curr. Dir. Psychol. Sci. 17, 313–317 10.1111/j.1467-8721.2008.00597.x

[B2] Atkinson J. Campbell F. W. Francis M. R. (1976). The magic number 4+/−0: a new look at visual numerosity judgements. Perception 5, 327–334 10.1068/p050335 980674

[B3] Badets A. Andres M. Di Luca S. Pesenti M. (2007). Number magnitude potentiates action judgements. Exp. Brain Res. 180, 525–534 10.1007/s00221-007-0870-y 17279382

[B4] Barsalou L. W. (2008). Grounded cognition. Annu. Rev. Psychol. 59, 617–645 17705682 10.1146/annurev.psych.59.103006.093639

[B5] Bender A. Beller S. (2012). Nature and culture of finger counting: diversity and representational effects of an embodied cognitive tool. Cognition 124, 156–182 10.1016/j.cognition.2012.05.005 22695379

[B6] Cohen Kadosh R. Walsh V. (2009). Numerical representation in the parietal lobes: abstract or not abstract? Behav. Brain. Sci. 32, 313–328 10.1017/S0140525X09990938 19712504

[B7] Daar M. Pratt J. (2008). Digits affect actions: the SNARC effect and response selection. Cortex 44, 400–405 18387571 10.1016/j.cortex.2007.12.003

[B8] Defever E. Sasanguie D. Vandewaetere M. Reynvoet B. (2012). What can the same-different task tell us about the development of magnitude representations? Acta Psychol. (Amst.) 140, 35–42 22426429 10.1016/j.actpsy.2012.02.005

[B9] Dehaene S. Bossini S. Giraux P. (1993). The mental representation of parity and number magnitude. J. Exp. Psychol. Gen. 122, 371–396

[B10] Dehaene S. Changeux J.-P. (1993). Development of elementary numerical abilities: a neuronal model. J. Cogn. Neurosci. 5, 390–407 10.1162/jocn.1993.5.4.390 23964915

[B11] Di Luca S. Granà A. Semenza C. Seron X. Pesenti M. (2006). Finger-digit compatibility in Arabic numeral processing. Q. J. Exp. Psychol. (Hove) 59, 1648–1663 10.1080/17470210500256839 16873114

[B12] Di Luca S. Pesenti M. (2008). Masked priming effect with canonical finger numeral configurations. Exp. Brain Res. 185, 27–39 17909768 10.1007/s00221-007-1132-8

[B13] Domahs F. Moeller K. Huber S. Willmes K. Nuerk H.-C. (2010). Embodied numerosity: implicit hand-based representations influence symbolic number processing across cultures. Cognition 116, 251–266 10.1016/j.cognition.2010.05.007 20513381

[B14] Elbert T. Pantev C. Wienbruch C. Rockstroh B. Taub E. (1995). Increased cortical representation of the fingers of the left hand in string players. Science 270, 305–307 10.1126/science.270.5234.305 7569982

[B15] Fischer M. H. (2008). Finger counting habits modulate spatial-numerical associations. Cortex 44, 386–392 18387569 10.1016/j.cortex.2007.08.004

[B16] Fischer M. H. Zwaan R. A. (2008). Embodied language: a review of the role of the motor system in language comprehension. Q. J. Exp. Psychol. (Hove) 61, 825–850 10.1080/17470210701623738 18470815

[B17] Gallace A. Tan H. Z. Spence C. (2008). Can tactile stimuli be subitised? An unresolved controversy within the literature on numerosity judgements. Perception 37, 782–800 18605150 10.1068/p5767

[B18] Gallistel C. Gelman R. (1992). Preverbal and verbal counting and computation. Cognition 44, 43–74 1511586 10.1016/0010-0277(92)90050-r

[B19] Glenberg A. M. Kaschak M. P. (2002). Grounding language in action. Psychon. Bull. Rev. 9, 558–565 10.3758/BF03196313 12412897

[B20] Kaufman E. L. Lord M. W. Reese T. W. Volkmann J. (1949). The discrimination of visual number. Am. J. Psychol. 62, 498–525 10.2307/1418556 15392567

[B21] Krause F. Lindemann O. (2012). Expyriment (Version 0.5.2) [Software]. Available at: http://www.expyriment.org

[B22] Lindemann O. Abolafia J. M. Girardi G. Bekkering H. (2007). Getting a grip on numbers: numerical magnitude priming in object grasping. J. Exp. Psychol. Hum. Percept. Perform. 33, 1400–1409 10.1037/0096-1523.33.6.1400 18085952

[B23] Lindemann O. Alipour A. Fischer M. H. (2011). Finger counting habits in middle eastern and western individuals: an online survey. J. Cross Cult. Psychol. 42, 566–578

[B24] Lindemann O. Krause F. (2012). Zählen mit den fingern: verkörperung oder veranschaulichung? Lernen Lernstörungen 1, 60–62

[B25] Lindemann O. Rueschemeyer S.-A. Bekkering H. (2009). Symbols in numbers: from numerals to magnitude information. Behav. Brain Sci. 32, 341–342 10.1017/S0140525X09990550

[B26] Loftus G. R. Masson M. E. J. (1994). Using confidence intervals in within-subject designs. Psychon. Bull. Rev. 1, 476–490 10.3758/BF03210951 24203555

[B27] Moeller K. Fischer U. Link T. Wasner M. Huber S. Cress U. (2012). Learning and development of embodied numerosity. Cogn. Process. 13, 271–274 10.1007/s10339-012-0457-9 22806653

[B28] Moyer R. S. Landauer T. K. (1967). Time required for judgements of numerical inequality. Nature 215, 1519–1520 10.1038/2151519a0 6052760

[B29] Nieder A. Miller E. K. (2003). Coding of cognitive magnitude: compressed scaling of numerical information in the primate prefrontal cortex. Neuron 37, 149–157 10.1016/S0896-6273(02)01144-3 12526780

[B30] Oliveri M. Vicario C. M. Salerno S. Koch G. Turriziani P. Mangano R. (2008). Perceiving numbers alters time perception. Neurosci. Lett. 438, 308–311 18486340 10.1016/j.neulet.2008.04.051

[B31] Piazza M. Izard V. Pinel P. Le Bihan D. Dehaene S. (2004). Tuning curves for approximate numerosity in the human intraparietal sulcus. Neuron 44, 547–555 15504333 10.1016/j.neuron.2004.10.014

[B32] Plaisier M. A. Bergmann Tiest W. M. Kappers A. M. L. (2009). One, two, three, many – subitizing in active touch. Acta Psychol. (Amst.) 131, 163–170 10.1016/j.actpsy.2009.04.003 19460685

[B33] Plaisier M. A. Smeets J. B. J. (2011). Haptic subitizing across the fingers. Atten. Percept. Psychophys. 73, 1579–1585 10.3758/s13414-011-0124-8 21479724 PMC3118010

[B34] Restle F. (1970). Speed of adding and comparing numbers. J. Exp. Psychol. 83(Pt 1), 274–278 10.1037/h0028573

[B35] Riggs K. J. Ferrand L. Lancelin D. Fryziel L. Dumur G. Simpson A. (2006). Subitizing in tactile perception. Psychol. Sci. 17, 271–272 10.1111/j.1467-9280.2006.01696.x 16623680

[B36] Tzelgov J. Meyer J. Henik A. (1992). Automatic and intentional processing of numerical information. J. Exp. Psychol. Learn. Mem. Cogn. 18, 166

[B37] van Ede F. Jensen O. Maris E. (2010). Tactile expectation modulates pre-stimulus beta-band oscillations in human sensorimotor cortex. Neuroimage 51, 867–876 10.1016/j.neuroimage.2010.02.053 20188186

[B38] Van Opstal F. Verguts T. (2011). The origins of the numerical distance effect: the same–different task. J. Cogn. Psychol. (Hove) 23, 112–120

[B39] Vicario C. M. (2012). Perceiving numbers affects the internal random movement generator. ScientificWorldJournal 2012, 6 10.1100/2012/347068 PMC3353301 22629133

[B40] Walsh V. (2003). A theory of magnitude: common cortical metrics of time, space and quantity. Trends Cogn. Sci. (Regul. Ed.) 7, 483–488 10.1016/j.tics.2003.09.002 14585444

[B41] Wilson M. (2002). Six views of embodied cognition. Psychon. Bull. Rev. 9, 625–636 10.3758/BF03196314 12613670

